# X-ray-induced Scintillation Governed by Energy Transfer Process in Glasses

**DOI:** 10.1038/s41598-017-18954-y

**Published:** 2018-01-12

**Authors:** Hirokazu Masai, Go Okada, Aya Torimoto, Takaaki Usui, Noriaki Kawaguchi, Takayuki Yanagida

**Affiliations:** 1 National Institute of Advanced Industrial Science and Technology, 1-8-31 Midorigaoka, Ikeda, Osaka 563-8577 Japan; 2 Nara Institute of Science and Technology, 8916-5 Takayama-cho, Ikoma, Nara 630-0192 Japan; 3 Institute for Chemical Research, Kyoto University, Gokasho, Uji, Kyoto, 611-0011 Japan

## Abstract

The efficiency of X-ray-induced scintillation in glasses roughly depends on both the effective atomic number *Z*_eff_ and the photoluminescence quantum efficiency *Q*_eff_ of glass, which are useful tools for searching high-performance phosphors. Here, we demonstrate that the energy transfer from host to activators is also an important factor for attaining high scintillation efficiency in Ce-doped oxide glasses. The scintillation intensity of glasses with coexisting fractions of Ce^3+^ and Ce^4+^ species is found to be higher than that of a pure-Ce^3+^-containing glass with a lower *Z*_eff_ value. Values of total attenuation of each sample indicate that there is a non-linear correlation between the scintillation intensity and the product of total attenuation and *Q*_eff_. The obtained results illustrate the difficulty in understanding the luminescence induced by ionizing radiation, including the energy absorption and subsequent energy transfer. Our findings may provide a new approach for synthesizing novel scintillators by tailoring the local structure.

## Introduction

Phosphors are a kind of energy converters that generate light in a broad range of wavelengths from ultraviolet (UV) to infrared (IR). Although most phosphors possess the ability to convert light^1–35^, some phosphors emit light as a result of mechanical stress^36,37^. Conventional phosphors are classified into two types: phosphors excited by UV or visible light^2–26^ and phosphors excited by ionizing radiation^21–35^. For the latter, the photon energy is far beyond the band gap of materials and radiation-induced luminescence is brought about by energy transfer from the host matrix to activators^38–41^. Therefore, X-ray-induced scintillation is a complex process involving absorption of X-rays in the matrix and scintillation in the activators. The absorption of X-rays by a material, i.e. the total attenuation of ionizing radiation, which can be expressed in terms of an absorption cross-section, is proportional to the density of the material, *ρ*, and the fourth-power of effective atomic number of the material, Z_eff_^4,38,39^. On the other hand, the scintillation efficiency, *η*, is typically expressed as *η* = *β*_e-h_·*S*_trans_·*Q*_eff_, where *β*_e-h_, *S*_trans_, and *Q*_eff_ are the efficiencies of the processes for generating electron-hole pairs (generation of the secondary particles), transferring the energies of the secondary particles to luminescent centres, and exciting and emitting light at luminescent centres, respectively. The value of *Q*_eff_ is conventionally referred to as the internal quantum efficiency of photoluminescence (PL). Generally, the development of scintillators mainly focuses on the values of *Z*_eff_ and *Q*_eff_ because it is difficult to discuss quantitatively the efficiencies for the electron-hole generation or energy transfer processes. This is the reason why most studies have been performed using lanthanide-doped garnet crystals.

On the other hand, our group has focused on amorphous materials. Owing to their wide chemical composition range and good formability, glasses can be good candidates for detection of ionizing radiation^27–29^. One of the glasses reported for phosphor applications is a Ce-doped lithium borosilicate glass^27^. Although this type of glass contains no heavy element, it is a good reference for the following reasons: (1) Since the glass can be prepared in an inert atmosphere, clear emission properties of Ce^3+^ are observed. (2) The *Q*_eff_ values of the glasses are sufficiently high to discuss the changes of the scintillation efficiency. (3) Both B_2_O_3_ and SiO_2_ can make glass networks, which correlates with the energy absorption and transfer process to the activators. (4) Valence states of Ce can be quantitatively discussed by using X-ray absorption near edge structure (XANES) analyses due to the lack of heavy cations whose absorption regions may overlap. A change in the compositional fraction of B_2_O_3_ and SiO_2_ is equivalent to a change in the value of *Z*_eff_. Therefore, it is worthwhile to examine the PL and scintillation properties of Ce^3+^ in this glass system.

The aim of this study is to investigate the relationship between the valence state of activators in the lithium borosilicate glasses possessing different *Z*_eff_ values and the PL and scintillation efficiency. In order to discuss the valence state of cerium, the *Ce*^3+^ *ratio* in the glasses is introduced and defined as the ratio of the Ce^3+^concentration to the sum of the concentrations for Ce^3+^ and Ce^4+^. Based on several analytical data, we have found that there is an anomalous relationship between the scintillation properties and the chemical composition of glass.

## Results

**Figure 1:**
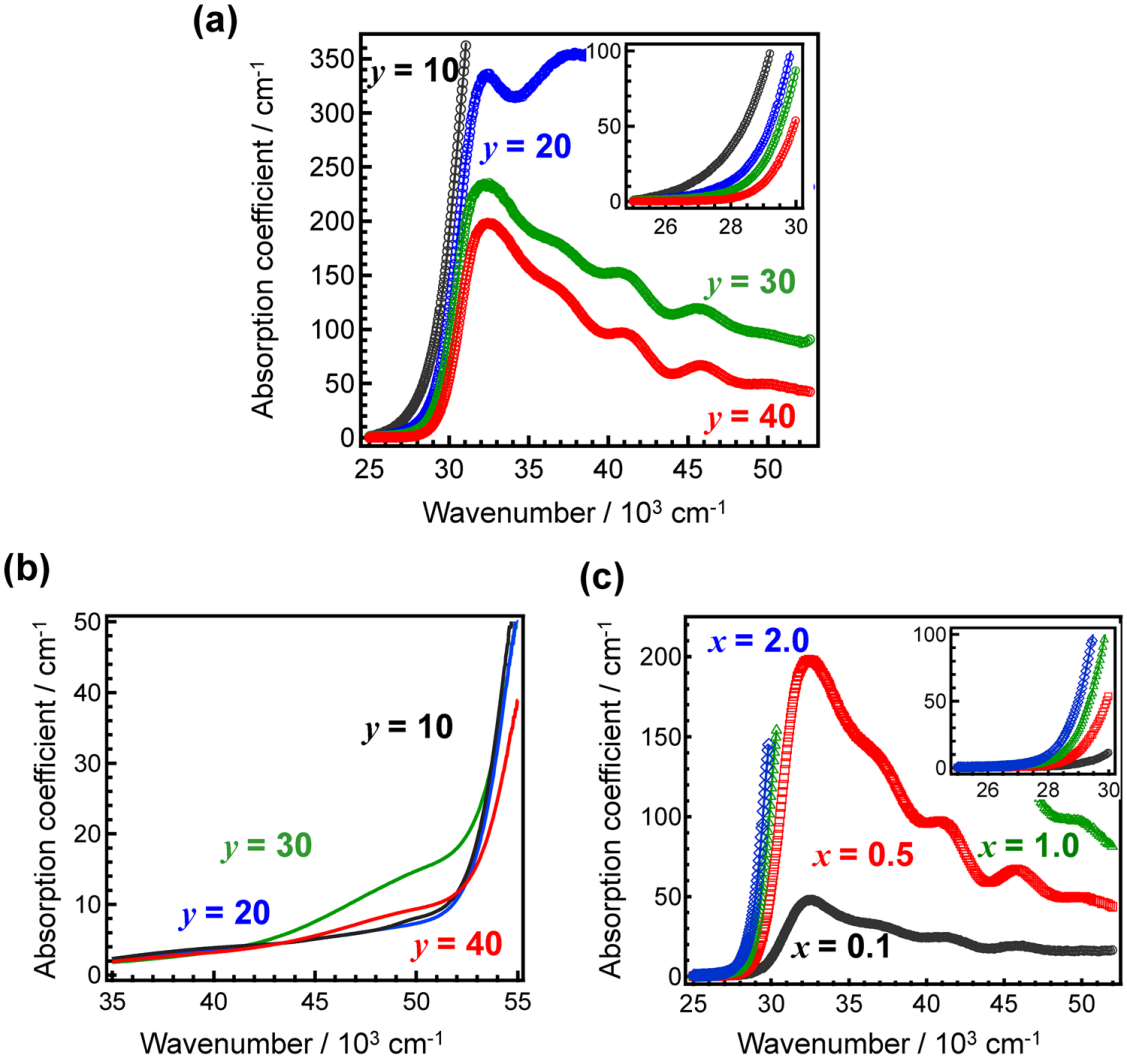
Optical absorption spectra of 0.5Ce:LBS*y* glasses. Optical absorption spectra of (**a**) 0.5Ce:LBS*y* glasses, (**b**) non-doped LBS*y* glasses and (**c**) *x*Ce:LBS40 glasses. The insets in Fig. 1(a) and (c) show zoomed-in view of the spectra at the optical absorption edge of these glasses.

The chemical composition of the present glass system is *x*Ce^3+^-40Li_2_O–*y*B_2_O_3_–(60-*y*)SiO_2_ (in molar ratio), where an excess amount of Ce is added. Herein, the general glass system is abbreviated as *x*Ce:LBS*y*. First, we examined several Ce-doped Li_2_O-B_2_O_3_-SiO_2_ glasses in order to change the *Z*_eff_ value. An increase in the amount of SiO_2_ increases the value of *Z*_eff_, which determines the effective absorption of X-ray energy. The chemical composition and the nominal *Z*_eff_ values of these glasses are listed in Table S1. Figure 1(a) shows the optical absorption spectra of 0.5Ce:LBS*y* glasses at room temperature (RT) for different values of *y*. Comparison of the absorption spectra for 0.5Ce:LBS*y* glasses with those of non-doped LBS*y* glasses (Fig. 1(b)) demonstrates that most of the absorption is due to the addition of Ce. Furthermore, the shape of the spectra in Fig. 1(a) changes considerably with the value of *y* (i.e. the B_2_O_3_-SiO_2_ ratio). On the other hand, Fig. 1(c) shows that the shape of the spectra slightly changes with the value of *x* (i.e. the Ce concentration)^27^. As shown in the inset of Fig. 1(c), when the chemical composition of LBS*y* is fixed, the optical absorption edge is slightly red-shifted with increasing amounts of Ce^3+^ due to be a broadening of the tail, i.e. a local coordination change (see Fig. S1). However, as shown in the inset of Fig. 1(a), the absorption coefficient at the tail region is largely red-shifted with increasing SiO_2_ fractions. Therefore, it is expected that the absorption shape depends on both parameters *x* and *y*. Since the absorption tail of Ce^4+^ is observed at low energy regions^28,29^, it is assumed that the red-shift of the absorption tail is correlated with the generation of Ce^4+^ species. Clear absorption bands are observed for LBS30 and LBS40 glasses. After a peak deconvolution using six absorption peaks with a half-width at half-maximum of approximately 2250 cm^−1^, we found that the photon energy of each excitation peak is almost the same. The results suggest that the Ce^3+^ coordination is almost the same for both glasses and that the activators are dispersed homogenously in the glass matrix.

**Figure 2:**
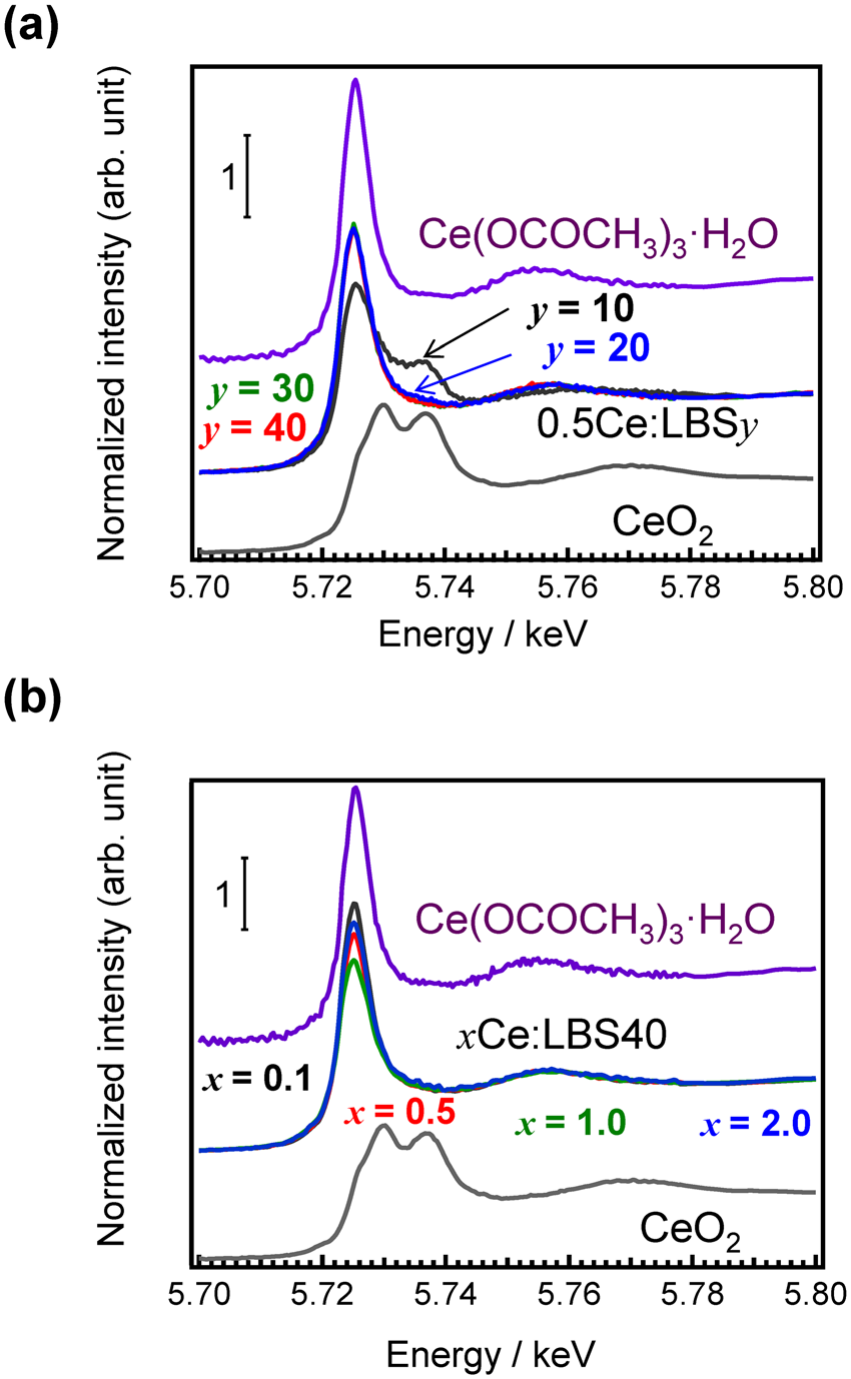
Ce XANES spectra of *x*Ce:LBS*y* glasses. Cerium L_III_-edge XANES analysis of (**a**) 0.5Ce:LBS*y* glasses and (**b**) the *x*Ce:LBS40 glasses along with data for Ce(OCOCH_3_)_3_·H_2_O and CeO_2_.

**Figure 3:**
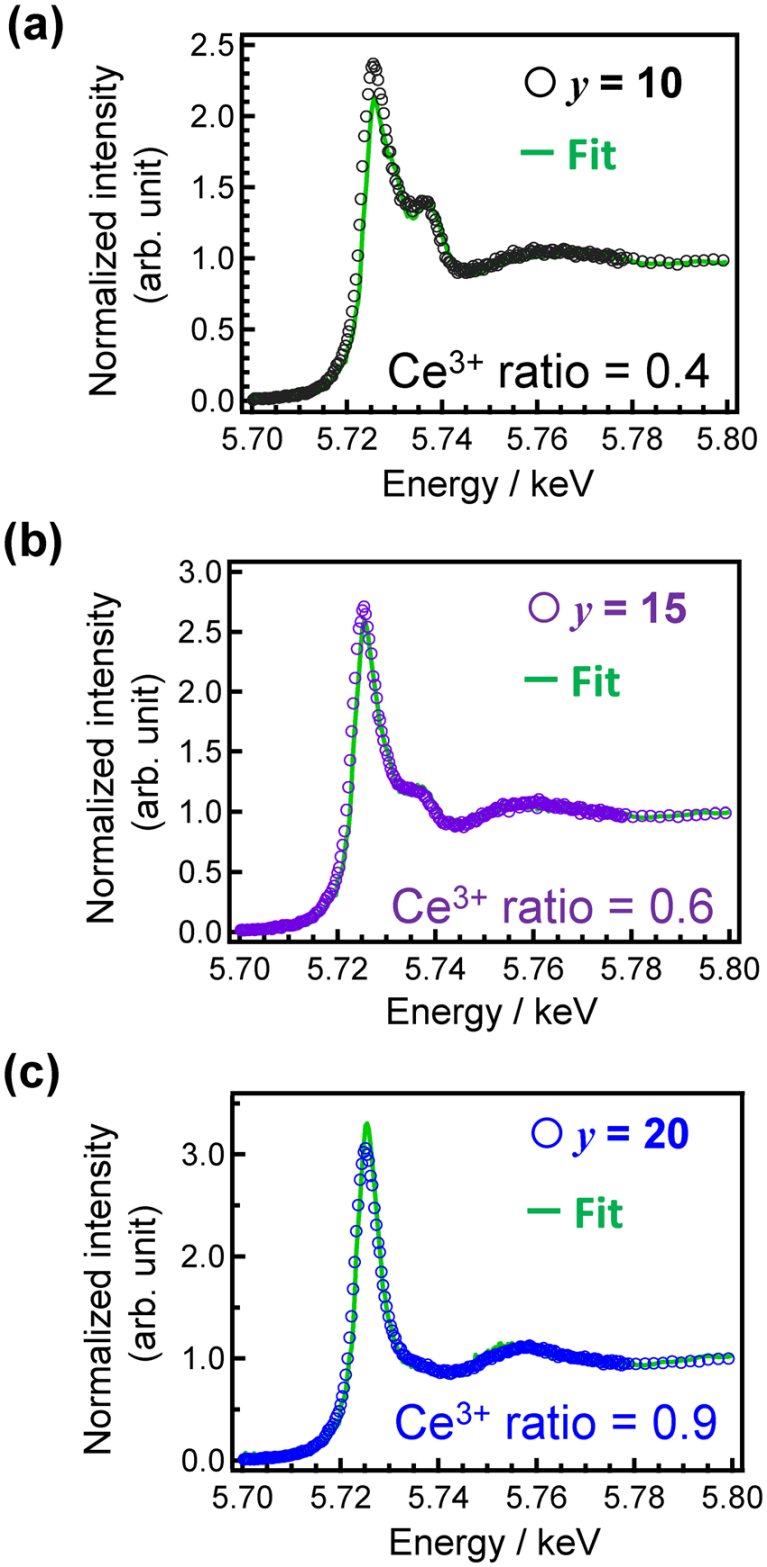
Cerium L_III_ XANES spectra of 0.5Ce:LBS*y* glasses along with fitting curves constructed by combination of XANES spectra of Ce(OCOCH_3_)_3_·H_2_O and CeO_2_.

In order to examine the valence state, we measured Ce L_III_-edge XANES spectra of 0.5Ce:LBS*y* glasses, as shown in Fig. 2(a). These white lines change with the B_2_O_3_-SiO_2_ ratio, especially near *y* = 10. The shape of the spectrum for the 0.5Ce:LBS40 glass is very similar to that of Ce(OCOCH_3_)_3_·H_2_O, as shown in Fig. S2, and noticeable differences for varying Ce concentrations are not observed (Fig. 2(b)). We can, therefore, conclude that the valence state of almost all (>95%) Ce centres in these LBS40 glasses are Ce^3+^ states, which is independent of the Ce concentration. Although precise fitting is difficult, the *Ce*^3+^ *ratio* of these glasses can be evaluated by spectra deconvolution using the spectra of Ce(OCOCH_3_)_3_·H_2_O and CeO_2_. Using these two reference materials, the *Ce*^3+^ *ratios* can be calculated as shown in Fig. 3. In the case of Ce:LBS30 and LBS40 glasses, the valence state of Ce is mostly the trivalent state. However, when the SiO_2_ fraction increases, the *Ce*^3+^ *ratio* decreases. It is notable that the XANES spectrum of the 0.5Ce:LBS10 glass is very similar to that of the 0.5Ce:LBS40 glass prepared in air (Fig S3), and that the *Ce*^3+^ *ratio* is less than 40 %, although the preparation of 0.5Ce:LBS10 was performed in an Ar atmosphere.

**Figure 4:**
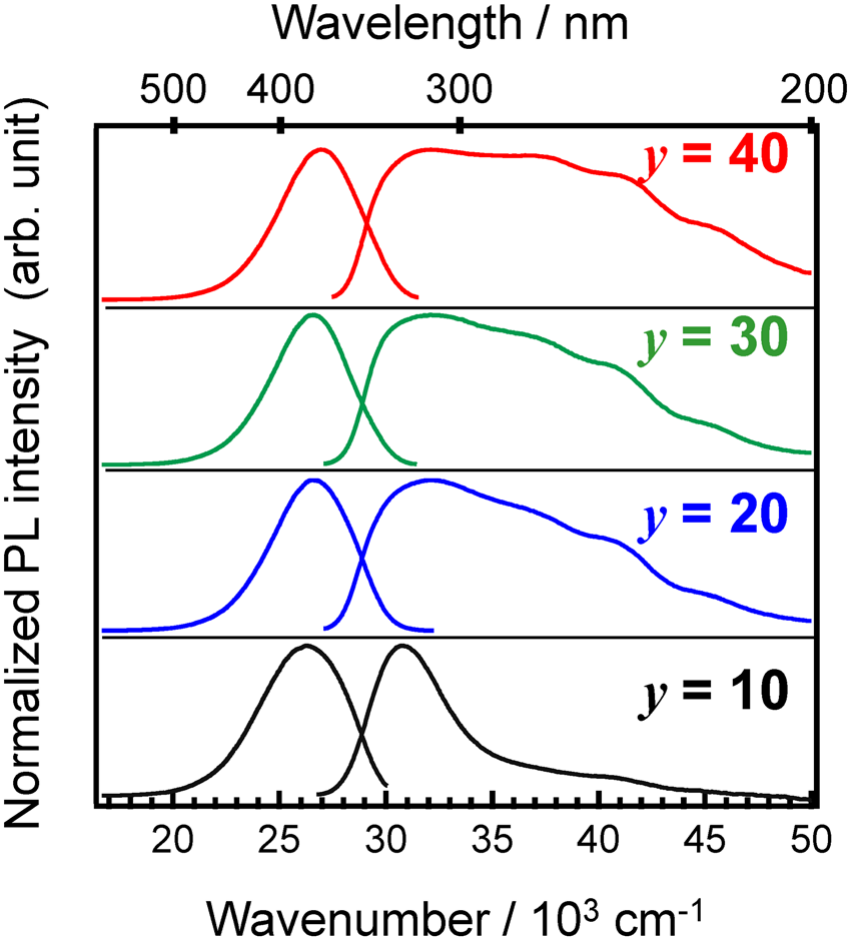
Photoluminescence-PLE spectra of 0.5Ce:LBS*y* glasses. The excitation and monitored wavenumbers correspond to the peaks of each spectrum.

**Figure 5:**
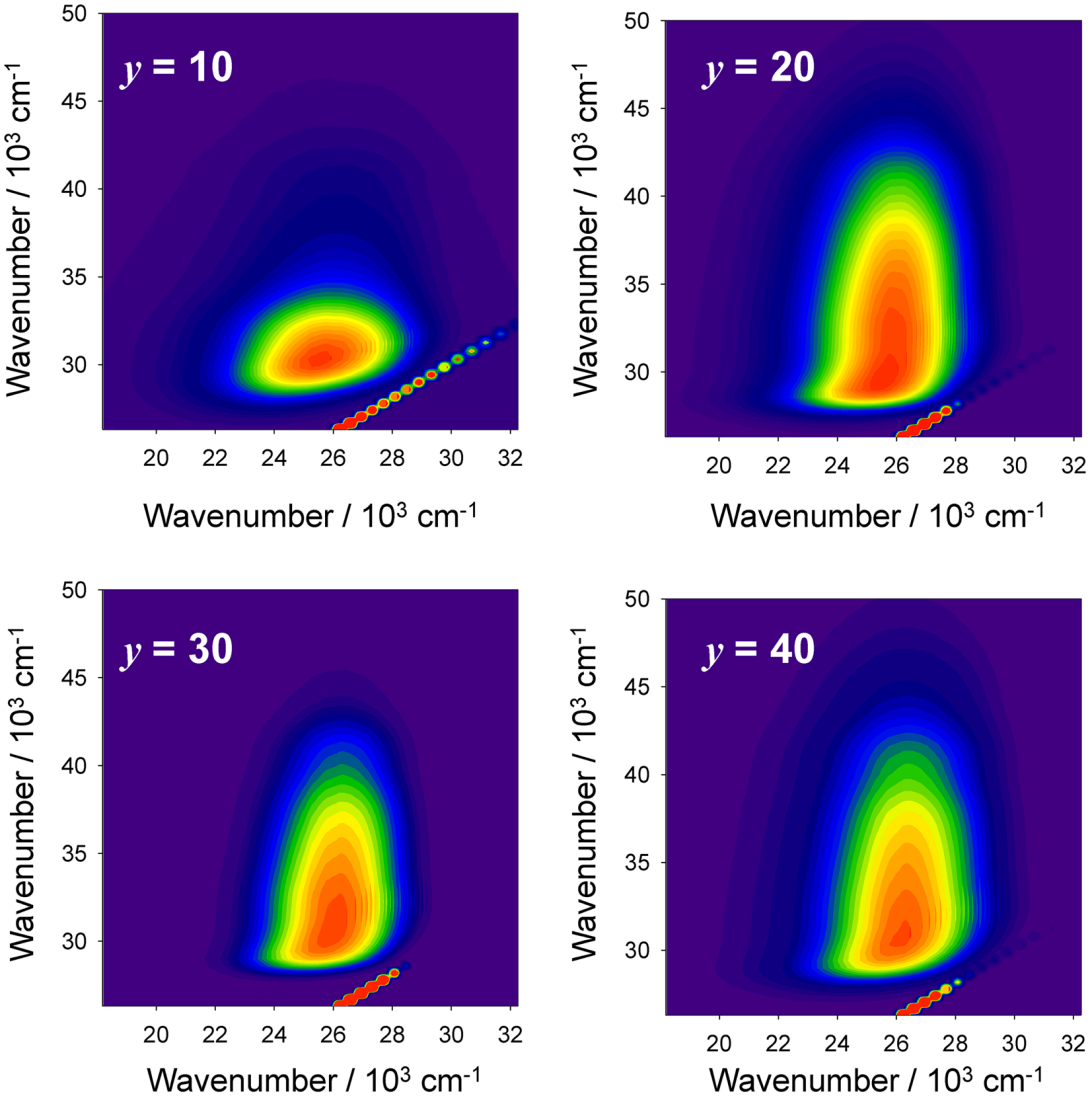
Contour plots of the PL-PLE spectra of 0.5Ce:LBS*y* glasses. The vertical and horizontal axes represent excitation and emission wavenumbers, respectively.

**Figure 6:**
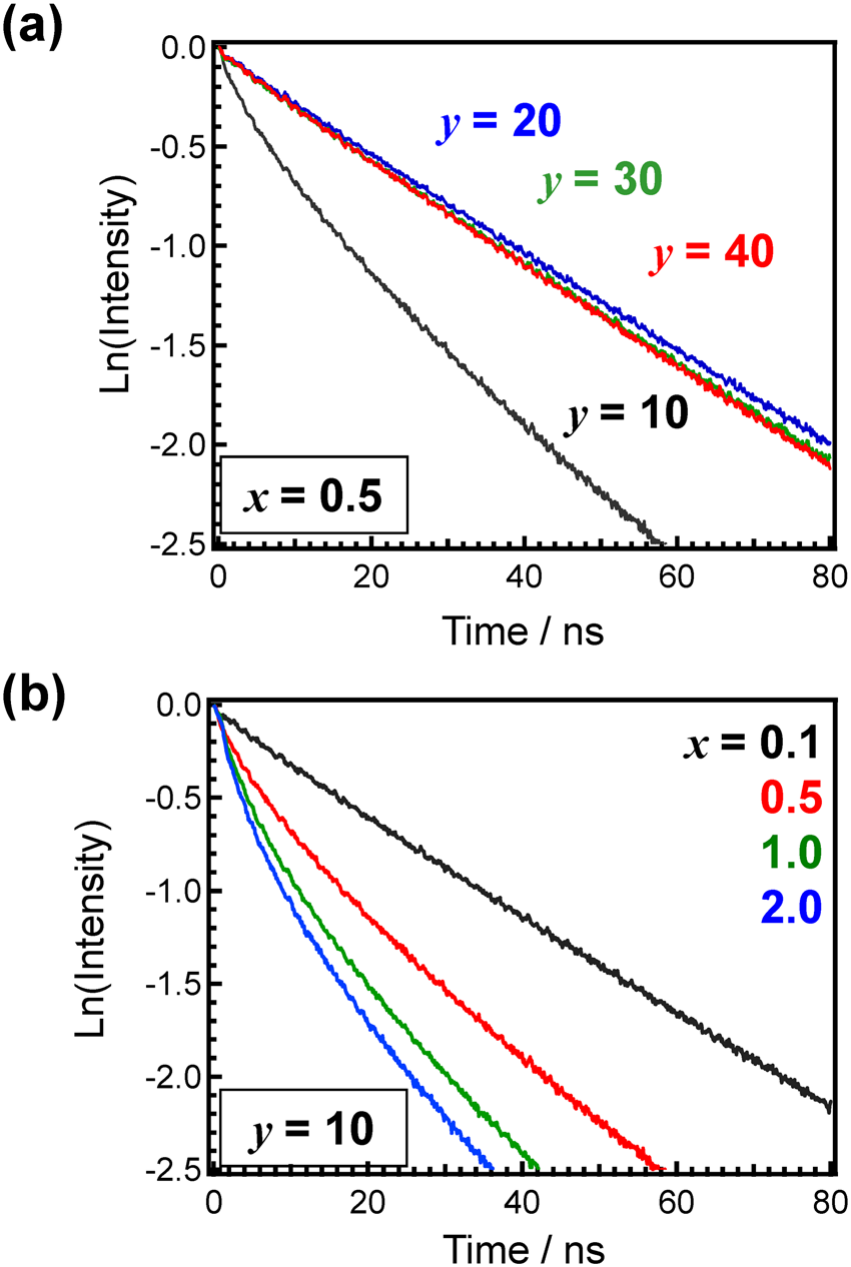
Photoluminescence decay curves of *x*Ce:LBS*y* glasses. Photoluminescence decay curves of (**a**) 0.5Ce:LBS*y* glasses and (**b**) *x*Ce:LBS10 glasses. The excitation and monitored wavenumbers (wavelengths) are 29,400 cm^−1^ (340 nm) and 25,000 cm^−1^ (400 nm), respectively.

**Figure 7:**
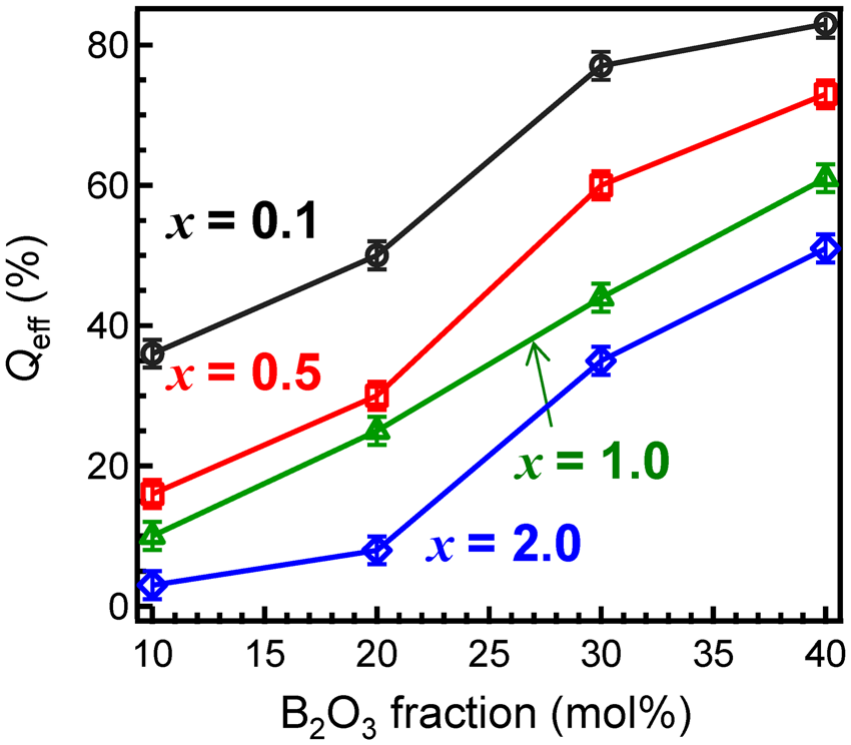
Quantum efficiency of *x*Ce:LBS*y* glasses as a function of the B_2_O_3_ fraction.

Figure 4 shows PL and PL excitation (PLE) spectra of 0.5Ce:LBS*y* glasses at RT. The wavenumbers of both the excitation and emission peaks of Ce^3+^ for the present glass are lower than those in phosphate glasses^19,20^ while higher than those in silicate glasses^20^. As the B_2_O_3_ fraction decreases, both peaks are slightly red-shifted, i.e. a smaller excitation energy induces a smaller emission energy. This might be correlated with the behaviour of the optical absorption spectra shown in Fig. 1(a), in which the absorption tail red-shifts with decreasing B_2_O_3_ fraction. Figure 5 shows contour plots of the PL-PLE spectra of 0.5Ce:LBS*y* glasses, where the PL intensity was normalized in order to understand the shapes of the spectra. The vertical and horizontal axes show the photon wavenumbers of excitation and emission, respectively. The fact that the excitation band is broad suggests that it is associated with the continuous excitation band, which is characteristic of Ce^3+^ states. However, as shown in Figs 4 and 5, the spectrum shape of the LBS10 glass is quite different from the shapes of the spectra for other B_2_O_3_ fractions. Irregularities associated with the LBS10 glass are also evident in the PL decay curves of *x*Ce:LBS*y* glasses shown in Fig. 6(a) for different B_2_O_3_ fractions. Specifically, a clear deviation from the linearity of the decay curves is observed for a B_2_O_3_ fraction of *y* = 10 shown in Fig. 6(b) and Fig S4. The decay constants of *x*Ce:LBS*y* glasses are summarized in Table S2. The internal quantum efficiencies *Q*_eff_ of *x*Ce:LBS*y* glasses are shown in Table S3 and Fig. 7. The values of *Q*_eff_ roughly depend on the Ce concentration and variations of *Q*_eff_ are probably due to differences in the local coordination state.

**Figure 8:**
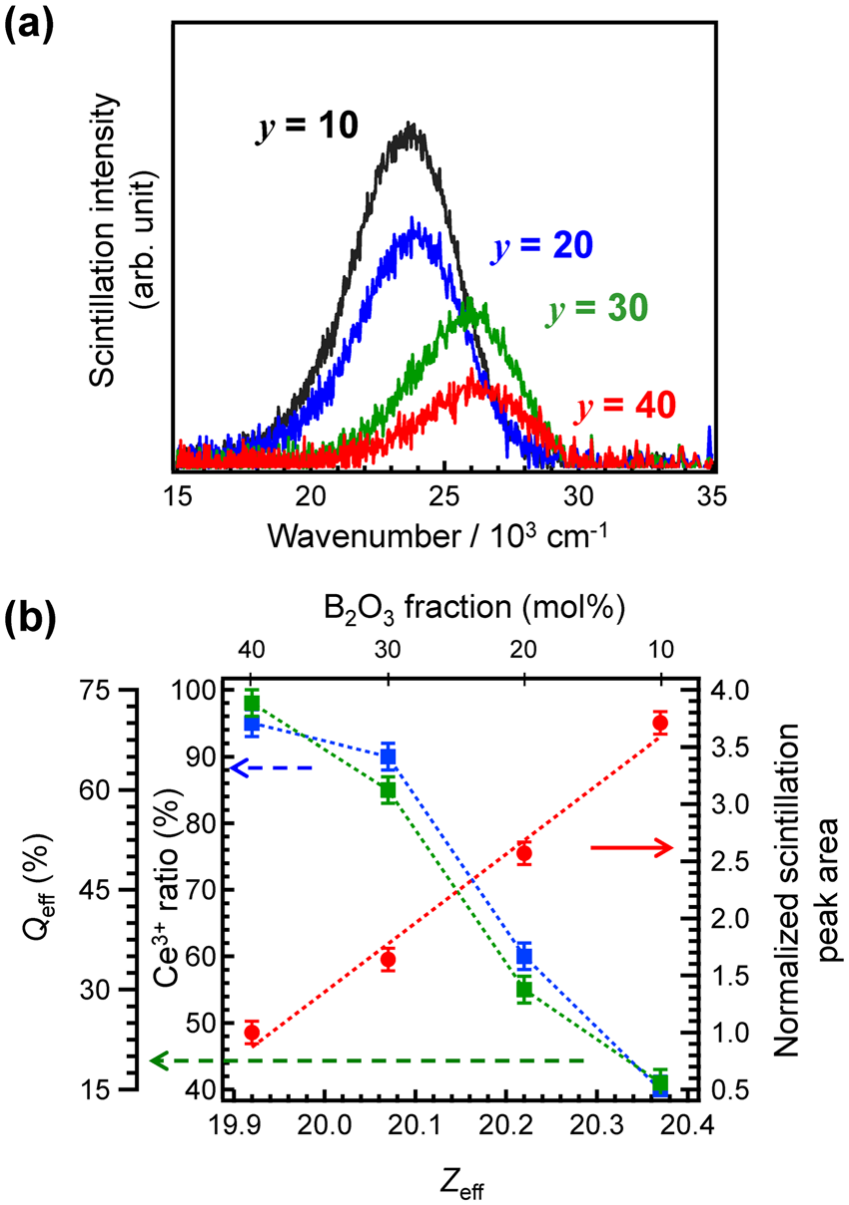
Scintillation properties of 0.5Ce:LBS*y* glasses. (**a**) X-ray-induced scintillation spectra of 0.5Ce:LBS*y* glasses by an irradiation dose of 10 Gy. (**b**) Values of *Q*_eff_, the *Ce*^*3+*^ *ratio*, and the normalized scintillation peak area as a function of *Z*_eff_ and the B_2_O_3_ fraction.

Figure 8(a) shows X-ray induced scintillation spectra of 0.5Ce:LBS*y* glasses obtained by and irradiation dose of 10 Gy. The scintillation intensities are normalized using the volume of the sample. We have confirmed that the scintillation spectra were unchanged during irradiation and that there is a linear relationship between the irradiation dose and the scintillation intensity (Fig. S5(a) and (b)). Figure 8(a) also shows that emission peak wavenumbers of Ce^3+^ red-shift with decreasing B_2_O_3_ fraction, as was observed in the PL spectra. It is noteworthy that the emission peak area of the 0.5Ce:LBS10 glass is much larger than that of the 0.5Ce:LBS40 glass, although we have confirmed that many Ce species are oxidized into Ce^4+^ during melting. In order to discuss the *Ce*^3+^ *ratio* quantitatively, the values of *Q*_eff_, and the scintillation peak area (normalized to the peak area of the 0.5Ce:LBS40 glass) are plotted in Fig. 8(b) as a function of *Z*_*eff*_ (bottom axis) and the B_2_O_3_ fraction (upper axis). It is evident that the scintillation intensity is proportional to *Z*_eff_ and inversely proportional to *Q*_eff_ and the *Ce*^3+^ *ratio*.

## Discussion

We have found that the chemical composition of glass affects the valence state of the activator in glasses. The results clearly suggest that the average *Ce*^3+^ *ratio* is affected by the chemical composition of glass, i.e. the macroscopic basicity of glass. In order to explain the results, we use the concept of the ‘optical basicity’ defined by Duffy^42,43^. Optical basicity, i.e. the average basicity of oxides in the glass, is a concept based on the polarization of electrons. The idea of basicity of glasses is sometimes useful for evaluation of the physical properties of bulk glasses. The optical basicity of Li_2_O, B_2_O_3_, and SiO_2_ are reported to be 1, 0.42, and 0.48, respectively^43^. Therefore, when the optical basicity of glass increases by substitution of SiO_2_ for B_2_O_3_, it is expected that an oxidation reaction of Ce^3+^ into Ce^4+^ occurs even in an Ar atmosphere. Since the starting materials of glass can affect the valence state of Ce cations^29^, it is not possible to reach a direct conclusion from the observed phenomena. However, an increase of the optical absorption in SiO_2_-rich glasses is expected to be brought about by a redox reaction transforming Ce^3+^ into Ce^4+^.

To the best of our knowledge, the physics of ionizing radiation is still unclear because of the complexity of the process. Therefore, research on scintillators is often conducted by focusing on specific parameters. Although *Q*_eff_ is generally a useful parameter to develop scintillators, *Z*_eff_ has been found to play a more dominant role for X-ray-induced scintillators^34^.

**Figure 9:**
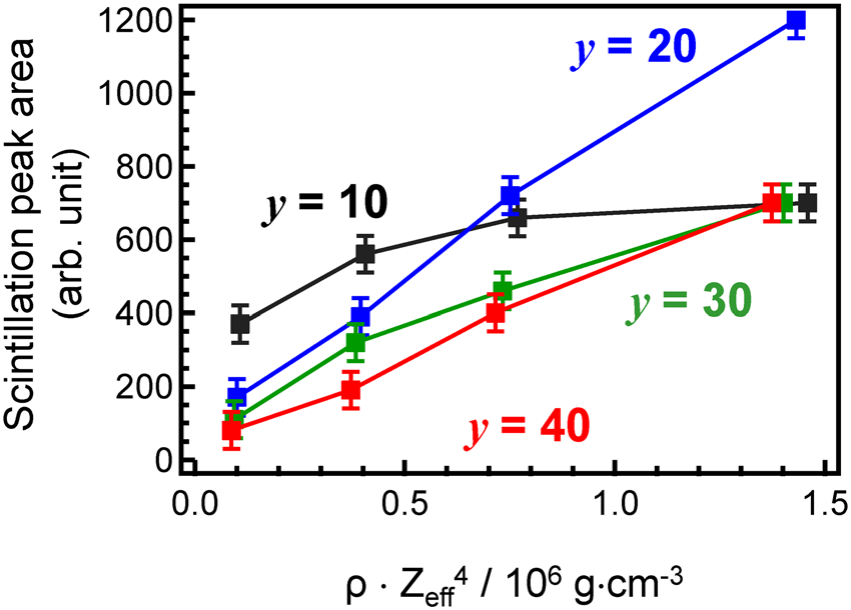
X-ray-induced scintillation peak area of *x*Ce:LBS*y* glasses as a function of ρ·Z_eff_^4^ for different B_2_O_3_ fractions.

As mentioned above, an increase in the SiO_2_ fraction causes an increase of *Z*_eff_, which in turn increases the effective absorption of X-rays. Figure 9 shows the X-ray-induced scintillation peak area of *x*Ce:LBS*y* glasses as a function of the product *ρ*·Z_eff_^4^. Since the dopant concentration is less than 2 mol%, the density of glass, *ρ*, shown in Table S4^44^ can be used for the discussion. With the exception of Ce:LBS10 glasses, in which a decrease in scintillation intensity is observed due to the strong self-absorption in the visible region, the scintillation peak areas are roughly dependent on the Ce concentration. Although the value of *Q*_eff_ for the 0.5Ce:LBS10 glass is much lower than that of the 0.5Ce:LBS40 glass because of the generation of Ce^4+^ species, the scintillation peak area of the 0.5Ce:LBS10 glass is higher than the peak areas of most 0.5Ce:LBS*y* glasses in Fig. S9.

**Figure 10:**
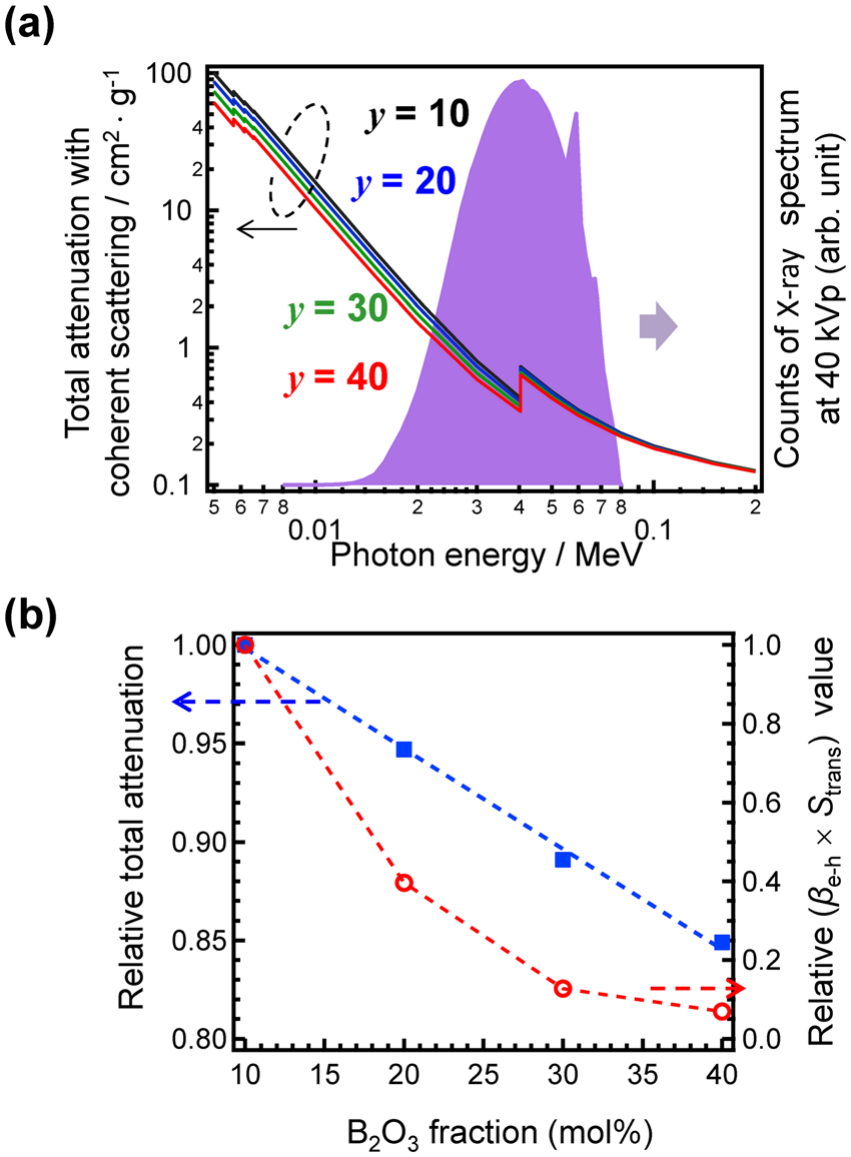
Total attenuation of 0.5Ce:LBS*y* glasses. (**a**) Total attenuation with coherent scattering of 0.5Ce:LBS*y* glasses. The scale of the spectrum of the tungsten lamp is given on the right y-axis. (**b**) Total attenuation and (*β*_e-h_ × *S*_trans_) relative to the values for the 0.5Ce:LBS10 glass.

Figure 10(a) shows the total attenuation with coherent scattering of 0.5Ce:LBS*y* glasses, which was calculated using a previously published fomula^45^ that takes into account the influence of *Z*_eff_. The energy spectrum of the X-rays used in the present study^46–48^ is also shown in the figure with a scale given on the right axis. Here, the X-ray source is a conventional X-ray tube with a W target and a Be window. In the energy region of irradiated X-rays, the total attenuation of the 0.5Ce:LBS10 glass is the highest among the present samples. Moreover, the attenuation values without coherent scattering exhibit a similar tendency. Here, we determined the total absorption energy using the following expression:

1 $$\zeta =\int E{N}_{0}(E)\frac{{\mu }_{{\rm{EA}}}(E)}{{\mu }_{{\rm{T}}}(E)}[1-\exp \{{\mu }_{T}(E)\cdot t\}]dE$$

where ζ is the absorbed energy in the sample along the irradiation axis per unit area, ***E*** is the incident radiation energy, ***N***_0_ is the number of incident photons per unit area, *μ*_T_(***E***) is the total attenuation coefficient of the sample, *μ*_*EA*_ is the energy absorption coefficient of sample, and *t* is the thickness of the sample.

Figure 10(b) shows the total absorption energy, ζ_relative_, relative to that of the 0.5Ce:LBS10 glass. In the present X-ray energy region, the value of ζ for the 0.5Ce:LBS10 glass is approximately 1.2 times larger than that of the 0.5Ce:LBS40 glass. As mentioned above, the scintillation intensity *I*_scinti_ is a product of the total absorption energy ζ and the scintillation efficiency *η* = *β*_e-h_·*S*_trans_·*Q*_eff_ and is given by

2 $${I}_{{\rm{scinti}}}=\zeta \,.\,{\beta }_{e-h}\,.\,{S}_{{\rm{trans}}}\,.\,{Q}_{{\rm{eff}}}$$

Since we have no quantitative information about the values of *β*_e-h_ and *S*_trans_, their product, (*β*_e-h_ .  *S*_trans_), is treated as a coefficient that can be evaluated using *I*_scinti_, ζ_relative_, and *Q*_eff_, and represents the efficiency for generating electron-hole pairs followed by energy transfer to luminescent centres in each glass. Using the values depicted in Figs 3(b) and 4(b), we have found that the value of (*β*_e-h_ .  *S*_trans_) for the 0.5Ce:LBS10 glass is more than 14 times larger than that of the 0.5Ce:LBS40 glass (see right axis of Fig. 4(b)). In other words, the absorbed X-ray energy is not converted into scintillation photons effectively in 0.5Ce:LBS40 glasses.

Plausible reasons for the low conversion efficiency are the physical parameters of non-doped LBS glasses shown in Table S4^44^. Since the molar volume of the LBS10 glass is smaller than that of the LBS40 glass, the network of the LBS10 glass is spatially denser than that of the LBS40 glass, i.e. there is larger free volume in the LBS40 glass. If there is no large difference in the phonon vibration energies of LBS*y* glasses, the free volume in the glasses may work as an attenuator and inhibit the effective energy transfer to activators. On the other hand, another reason for the low conversion efficiency is the storage mechanism of irradiated energy proposed by Yanagida^34^. It was reported that a B_2_O_3_-containing glass exhibits storage luminescence by X-ray irradiation^35^. Because the irradiated energy is converted into scintillation, storage luminescence, or thermal vibration (non-radiative relaxation), high storage luminescence means low scintillation. Considering that the origin of storage luminescence is defects in glasses, we speculate that there are many defects that affect the energy transfer process to activators in B_2_O_3_-rich glass. As shown in Fig. S6 and Table S5, there are only small differences in the band gaps for LBS*y* glasses and these differences cannot provide a plausible explanation for changes in the conversion efficiencies.

Recent studies have suggested that the fraction of Ce^4+^ in scintillators has an effect on scintillation properties^49–52^ and several of them claimed that coexistence of Ce^3+^ and Ce^4+^ is important for high scintillation efficiency^49–51^. However, if the coexistence of Ce^3+^ and Ce^4+^ was a critical factor for determining the intensity, the correlation between chemical composition and scintillation intensity, as shown in Fig. 3(a), would be quite different; i.e. Ce:LBS*y* glasses would exhibit similar intensities with the exception of the Ce:LBS10 glass. Therefore, the present results do not support the hypothesis that coexistence of Ce^3+^ and Ce^4+^ is important for high scintillation efficiency, at least in the present glass system. In turn, this work shows that the energy transfer process of the generated charged secondary particles to activators is important for attaining high scintillation efficiency. Therefore, tailoring the energy transfer process is expected to enable fabrication of high-performance scintillators.

## Conclusion

We have examined PL and X-ray-induced scintillation properties of several Ce-doped lithium borosilicate glasses. It was confirmed that only Ce^3+^ valence states exist in Ce:LBS40 glasses and that the *Ce*^*3*+^ *ratio* decreases with increasing SiO_2_ fraction in the glasses. The oxidation reaction in the glass melt in an inert atmosphere can be explained by the optical basicity of the glass, and the amount of Ce^4+^ generated is the origin of the absorption tail in the visible region of the absorption spectra. Although the value of *Q*_eff_ for the Ce:LBS10 glass is the smallest among all *Q*_eff_ values for the present LBS glasses, the scintillation intensity of the Ce:LBS10 glass is the highest because it has the highest attenuation values. In terms of the emission mechanism of scintillators, the effective energy conversion after absorbing the ionizing radiation is prevented in the B_2_O_3_-rich glasses. Such energy transfer path will be important for further materials design of radiation detectors.

## Methods

### Preparation of Ce-doped lithium borosilicate glass

The *x*Ce^3+^-40Li_2_O–*x*B_2_O_3_–(60-*y*)SiO_2_ (*x*Ce:LBS*y*) glasses were prepared according to a conventional melt-quenching method by employing a platinum crucible^24^. A mixture of Li_2_CO_3_ (99.99%), B_2_O_3_ (99.9%), SiO_2_ (99.999%), and Ce(OCOCH_3_)_3_·2H_2_O (99.9%) was melted in an electric furnace at 1100°C for 30 min in an Ar atmosphere (99.999%). The glass melt was quenched on a stainless plate at 200°C and then annealed at a temperature *T*_g_, which was measured by differential thermal analysis (DTA) for 1 h. The bulk glasses were cut into several glass pieces (10 mm × 10 mm) using a cutting machine, and then, samples were mechanically polished (thickness ~ 1 mm) to obtain mirror surfaces. The temperature *T*_g_ was determined by a DTA system operating at a heating rate of 10 °C/min using a TG8120 instrument (Rigaku, Japan). The density of the samples was measured using the Archimedes method with pure water as an immersion liquid.

### Luminescence properties

The PL and PLE spectra were recorded at 1 nm intervals at RT using an F7000 fluorescence spectrophotometer (Hitachi High-Tech. Japan). Band pass filters of 2.5 nm for the PL measurement were used for both excitation and emission. The absorption spectra at RT were recorded at 1 nm intervals using a U3500 UV-vis-NIR spectrometer (Hitachi High-Tech. Japan). The absolute quantum efficiencies, also known as quantum yields (QYs), of the glasses were measured using an integrating sphere Quantaurus-QY (Hamamatsu Photonics, Japan). The error bars were ±2. The emission decay at RT was measured using a Quantaurus-Tau system (Hamamatsu Photonics, Japan) with a 340 nm LED. The accumulated counts for evaluation were 50,000. Scintillation (radioluminescence) spectra were measured by using a CCD-based spectrometer (Andor DU920P CCD and SR163 monochromator) under X-ray exposure^23^. The supplied bias voltage and tube current were 40 kV and 0.52 ~ 5.2 mA, respectively.

### XANES measurement

The Ce L_III_-edge XANES spectra were measured at the BL01B1 and BL14B2 beamlines of SPring-8 (Hyogo, Japan). The storage ring energy was operated at 8 GeV with a typical current of 100 mA. The measurements were performed using a Si (111) double-crystal monochromator in the transmission mode (Quick Scan method), or in the fluorescence mode using 19-SSD detector at RT. The XANES spectra were recorded from 5.52 to 6.18 keV. Pellet samples for the measurements were prepared by mixing the granular sample with boron nitride. As references, XANES data for Ce(OCOCH_3_)_3_·2H_2_O and CeO_2_ were collected using the same conditions. The corresponding analyses were performed by using Athena software^53^.

## Electronic supplementary material

Supplementary Data
