# Supplementary material for: X-ray-induced Scintillation Governed by Energy Transfer Process in Glasses
Source: Sci Rep. 2018 Jan 12;8:623. doi: 10.1038/s41598-017-18954-y (PMC5766489; doi:10.1038/s41598-017-18954-y)
Supplement: Supplementary file 1 — Supplementary Data [file 41598_2017_18954_MOESM1_ESM.doc]

**X-ray-induced Scintillation Governed by Energy Transfer Process in Glasses**

by H. Masai, G. Okada, A. Torimoto, T. Usui, N. Kawaguchi, and T. Yanagida

**Supplementary Data**

**Supplementary Table 1 | Nominal Z_eff_ values of the *x*Ce-LBS*y* glasses**

| B_2_O_3_ Fraction,  *y* (mol%) | Ce concentration, ***x*** (mol%) | | | |
| --- | --- | --- | --- | --- |
|  | 0.1 | 0.5 | 1.0 | 2.0 |
| 10 | 14.61 | 20.38 | 23.89 | 28.05 |
| 20 | 14.37 | 20.22 | 23.75 | 27.91 |
| 30 | 14.14 | 20.07 | 23.61 | 27.76 |
| 40 | 13.90 | 19.92 | 23.47 | 27.61 |

**Supplementary Figure 1 |** Normalized absorbance of the *x*Ce:LBS*y* glasses.

**Supplementary Figure 2 |** Comparison of Ce L_III_-edge XANES spectra of the 0.5Ce:LBS40 glass along with Ce(OCOCH_3_)_3_·H_2_O and CeO_2_.

**Supplementary Figure 3 |** Ce L_III_-edge XANES spectra of the 0.5CeLBS*y* glasses along with that of Ce(OCOCH_3_)_3_·H_2_O. Ce L_III_-edge XANES spectrum of the 0.5CeLBS40 glass prepared in air is shown for comparison.

**Supplementary Figure 4 |** PL decay curves of ***x***Ce:LBS*y* glasses. The excitation energy was 29,400 cm^–1^ (340 nm) .

**Supplementary Table 2 | PL decay constants of the *x*Ce-LBS*y* glasses**

| B_2_O_3_ Fraction,  *y* (mol%) | Ce concentration, ***x*** (mol%) | | | |
| --- | --- | --- | --- | --- |
|  | 0.1 | 0.5 | 1.0 | 2.0 |
| 10 | 37±1 | 5, 28±1 | 5, 25±1 | 5, 23±1 |
| 20 | 37±1 | 41±1 | 38±1 | 37±1 |
| 30 | 36±1 | 39±1 | 37±1 | 36±1 |
| 40 | 36±1 | 39±1 | 37±1 | 35±1 |

**Supplementary Table 3 | Internal Quantum efficiency of *x*Ce:LBS*y* glasses. The error bars were ±2.**

| B_2_O_3_ Fraction,  *y* (mol%) | Ce concentration, ***x*** (mol%) | | | |
| --- | --- | --- | --- | --- |
|  | 0.1 | 0.5 | 1 | 2 |
| 10 | 36 | 16 | 10 | 3 |
| 20 | 50 | 30 | 25 | 8 |
| 30 | 77 | 60 | 44 | 35 |
| 40 | 83 | 73 | 61 | 51 |

**Supplementary Figure 5 |** (a) X-ray-induced scintillation spectra of 0.1Ce:LBS10 glasses obtained by irradiation of different doses. (b) Correlation between scintillation intensity and the irradiated doses of 0.1Ce:LBS10 glasses.

**Supplementary Table 4 | Physical parameters of non-doped LBS*y* glasses^44^.**

| B_2_O_3_ Fraction, ,*y* (mol%) | 10 | 20 | 30 | 40 |
| --- | --- | --- | --- | --- |
| Glass transition temperature / K | 720 | 727 | 730 | 737 |
| Melt temperature / K | 1070 | 1064 | 1059 | 1060 |
| Density / g⋅cm^–3^ | 2.36 | 2.36 | 2.35 | 2.35 |
| Molar volume/ cm^3^· mol^−1^ | 20.7 | 21.1 | 21.6 | 22 |
| Refractive index at 532 nm | 1.5638 | 1.5684 | 1.5713 | 1.5750 |
| Longitudinal sound velocity *V*_L_ / m· s^−1^ | 6815 | 6963 | 7016 | 7158 |
| Elastic modulus *c*_11_ / GPa | 110.1 | 114.9 | 116.2 | 120.4 |

**Supplementary Figure 6 |** Optical absorption spectra of LBS*y* glasses. In order to obtain the optical band gap, the vertical axis is (αhν)^1/2^, which is generally used for indirect transitions. (*α*, *h*, and *ν* are the absorption coefficient, the Planck constant, and the frequency, respectively.)

**Supplementary Table 5 | Optical band gap of non-doped LBS*y* glasses. The band gaps are estimated from extrapolation of their Tauc plots.**

| B_2_O_3_ Fraction, *y* (mol%) | 10 | 20 | 30 | 40 |
| --- | --- | --- | --- | --- |
| Optical band gap / 10^3^ cm^–1^ | 50.5 | 50.4 | 50.0 | 50.1 |
| Error bars / 10^3^ cm^–1^ | 0.3 | 0.3 | 0.4 | 0.4 |
